# Effect of Tryptic Digestion on Sensitivity and Specificity in MALDI-TOF-Based Molecular Diagnostics through Machine Learning

**DOI:** 10.3390/s23198042

**Published:** 2023-09-23

**Authors:** Sumon Sarkar, Abigail Squire, Hanin Diab, Md. Kaisar Rahman, Angela Perdomo, Babafela Awosile, Alexandra Calle, Jonathan Thompson

**Affiliations:** School of Veterinary Medicine, Texas Tech University, 7671 Evans Dr., Amarillo, TX 79106, USA; sumon.sarkar@ttu.edu (S.S.); absquire@ttu.edu (A.S.); kaisar.rahman@ttu.edu (M.K.R.);

**Keywords:** machine learning, molecular diagnostics, precision medicine, biomarker, MALDI-TOF, diagnostic, protein digest, artificial intelligence (AI)

## Abstract

The digestion of protein into peptide fragments reduces the size and complexity of protein molecules. Peptide fragments can be analyzed with higher sensitivity (often > 10^2^ fold) and resolution using MALDI-TOF mass spectrometers, leading to improved pattern recognition by common machine learning algorithms. In turn, enhanced sensitivity and specificity for bacterial sorting and/or disease diagnosis may be obtained. To test this hypothesis, four exemplar case studies have been pursued in which samples are sorted into dichotomous groups by machine learning (ML) software based on MALDI-TOF spectra. Samples were analyzed in ‘intact’ mode in which the proteins present in the sample were not digested with protease prior to MALDI-TOF analysis and separately after the standard overnight tryptic digestion of the same samples. For each case, sensitivity (sens), specificity (spc), and the Youdin index (J) were used to assess the ML model performance. The proteolytic digestion of samples prior to MALDI-TOF analysis substantially enhanced the sensitivity and specificity of dichotomous sorting. Two exceptions were when substantial differences in chemical composition between the samples were present and, in such cases, both ‘intact’ and ‘digested’ protocols performed similarly. The results suggest proteolytic digestion prior to analysis can improve sorting in MALDI/ML-based workflows and may enable improved biomarker discovery. However, when samples are easily distinguishable protein digestion is not necessary to obtain useful diagnostic results.

## 1. Introduction

The realm of diagnostic sensing technologies has undergone revolutionary transformations in recent decades, leading to significant advancements in precision medicine and personalized patient care. Among the various diagnostic tools, Matrix-Assisted Laser Desorption/Ionization Time-of-Flight Mass Spectrometry (MALDI-TOF MS) has emerged as a powerful technique for bacterial identification and molecular-based diagnostics [1,2,3,4,5]. Its ability to rapidly analyze complex biomolecules, such as intact proteins with minimal sample preparation, has revolutionized clinical diagnostics, enabling faster and more accurate sorting of bacterial species and disease states. One initial application of MALDI-TOF MS focused on creating a sensor for bacterial identification, where specific protein profiles are used to differentiate bacterial species [6,7,8,9,10,11,12,13,14]. The simplicity, speed, and accuracy of this method have revolutionized microbial identification in clinical settings. In recent years, this approach has been extended to molecular diagnostics, encompassing the identification of specific biomarkers associated with diseases, such as cancer and infectious diseases [15,16,17,18,19,20]. 

Given the potential in disease diagnosis and biomarker discovery, coupled MALDI-TOF plus machine learning (ML) workflows have been gaining significant attention [20,21,22,23,24]. Machine learning techniques have emerged as a powerful tool for uncovering patterns in complex data sets, including mass spectra obtained through MALDI-TOF MS analysis. By applying various algorithms, machine learning can decipher hidden information present in signals and improve classification accuracy. The integration of machine learning algorithms with MALDI-TOF-MS has introduced a paradigm shift in diagnostic approaches. By processing and analyzing vast datasets from mass spectra, machine learning algorithms can distinguish subtle differences between bacterial species and identify specific biomarkers associated with diseases. The impact of machine learning on disease diagnosis and biomarker discovery has already been transformative in healthcare and advances continue to rapidly progress [25,26,27,28,29,30,31]. However, to fully exploit the potential of MALDI-TOF MS in disease diagnosis, innovative approaches must continue to be explored to improve the sensitivity and specificity of diagnosis.

In this communication, we report that significant enhancement of dichotomous sorting can be achieved by a simple and well-known modification to typical MALDI workflows. The traditional analysis of intact proteins has been augmented using tryptic digestion, which breaks down proteins into smaller peptide fragments of lower mass [32,33,34]. In traditional MALDI-TOF-MS, intact proteins are predominantly analyzed for bacterial identification and disease diagnostics. However, the size of intact proteins can limit the resolution and, more crucially, the sensitivity of the mass spectrometer, potentially leading to challenges in accurately measuring low-abundance biomolecules. Tryptic digestion, a well-established technique in proteomics, involves the enzymatic cleavage of intact proteins into smaller peptide fragments of lower mass. Meanwhile, protein digestion itself is certainly not novel; herein, we provide the initial report of this procedural enhancement boosting both the sensitivity and specificity of MALDI-TOF-MS-based molecular diagnostics utilizing machine learning. We argue that gain in performance originates from the inherent sensitivity increase associated with the analysis of lower molecular weight fragments (compared to intact protein) and the ability to achieve sensitive, high-resolution reflectron mode measurements at masses < 3 kDa, the range of the mass spectrum within which tryptic fragments generally appear. Since smaller peptide fragments are analyzed, this increases the probability of detecting disease-specific biomarkers, which may be present at low concentrations in clinical samples and otherwise evade detection. Gains in the sorting performance of machine learning algorithms are shown to be of the highest value when samples are the most chemically similar. In the subsequent sections of this manuscript, we will describe four case studies that demonstrate the effectiveness of protein digestion for the dichotomous sorting of samples by machine learning.

## 2. Materials and Methods

**Figure 1:**
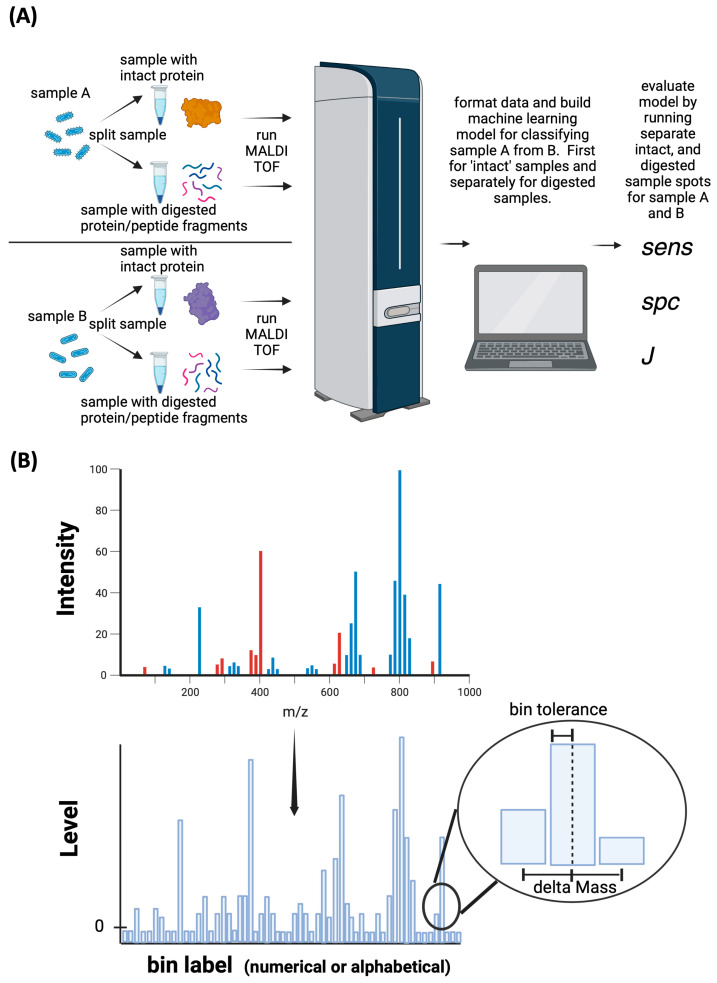
(**A**) Experimental workflow. Sample A and Sample B are the two sample groups/types we differentiate by the MALDI-TOF spectra. Intact protein samples and digested samples were analyzed independently by MALDI-TOF and spectra imported into machine-learning software. Sens, spc, and J were computed by Equations (1)–(3) to assess model performance for a separate scoring data set. (**B**) Illustrates the process of binning the MALDI-TOF spectra. See Section 2.6 of the text for details.

To investigate the effect of protein digestion we have conducted four case studies. For each case study, we have analyzed samples both 1) after tryptic digestion (digested) and 2) undigested (intact) by MALDI-TOF mass spectrometry, as summarized in Figure 1. After collecting mass spectra, peak lists were exported into machine learning software (RapidMiner) to train a machine learning model. A separate set of sample spots was also analyzed with the same workflow and used as a scoring dataset to evaluate the performance of the machine learning model. It should be noted the machine learning approach only detects differences/patterns in the MALDI-TOF spectra between data sets. The approach does not inherently detect biomarkers and, rather, only detects patterns apparent in data resulting from any chemical or physical reality. 

The performance of machine learning models was evaluated by computing the sensitivity (sens), specificity (spc), and Youden index (J) by Equations (1)–(3):

(1) $$sens=\frac{\#\;true\;positives\;identified}{total\;number\;of\;true\;positive\;samples\;}$$

(2) $$spc=\frac{\#\;true\;negatives\;identified}{total\;number\;of\;true\;negative\;samples}$$

J = sens + spc − 1(3)

Sensitivity is essentially the fraction of true positives detected. Specificity is the fraction of true negatives detected. Both high sensitivity and specificity are required for successful diagnostic tests; the Youden index (J) combines parameters to create a single parameter describing performance.

### 2.1. Case Study 1: Pancake Mix—Gluten-Free vs. Wheat Gluten Mix

#### Sample Preparation

Two pancake mixes were acquired from a market. The first was a gluten-free option and the second was a pancake mix with wheat flour gluten. However, it should be noted there were additional chemical differences between the samples. To prepare the samples for MALDI analysis, we used the protein extraction method of Meredith and Wren [35]. We began by adding 0.6 g of each mix into separate 15 mL Falcon tubes and diluting the mix in each tube to a volume of 14 mL, using the recommended solvent that is efficient in wheat protein extraction. The solvent was composed of 3 M urea, 0.01 M tetradecyl trimethyl ammonium bromide (TTAB), and 0.1 M acetic acid. We then mixed the contents of each Falcon tube vigorously with inversion. Once mixed, the tubes were centrifuged for 2 min at 3900 rpm to separate the undissolved mix from the supernatant. The supernatant was then collected, yielding a turbid colorless liquid. 

To make the undigested, or intact, samples, 0.5 mL of pancake extract solution (supernatant) from each mix was mixed with 1 mL of 33% ethanol/33% H_2_O/33% acetonitrile in 0.1% formic acid and this mixture was spotted onto MALDI plates. The machine learning model training data for the ‘intact’ sample set consisted of 48 samples of wheat gluten pancake mix and an additional 48 samples of gluten-free mix spotted onto the MALDI targets. The scoring/evaluation data set consisted of an additional 96 spots (48 gluten-free and 48 with wheat gluten). Thus, there were 192 total spots for the MALDI analysis. Each spot was composed of 1 μL of sample and 1 mL of CHCA matrix solution.

The digested samples were prepared by combining 0.5 mL of supernatant from both samples with 0.5 mL of bicarbonate buffer (pH = 7.9), with 0.2% SDS and 0.1 mM DTT, inside two 2 mL centrifuge tubes. Both centrifuge tubes were heated at 95 °C for 15 min to promote protein denaturation and dithiol bond reduction. Samples were then removed from heat and allowed to cool to room temp when an additional 1 mL of bicarbonate buffer/SDS solution and 50 μL of a 50 mg/mL iodoacetamide were added to each tube. Samples were incubated at room temperature for 30 min to alkylate thiols. Then, 50 μL of a 0.2 μg/μL solution of trypsin was added to each vial. Once mixed thoroughly, the tubes were incubated at 37 °C for 20 h. Finally, after the incubation process was completed, 0.1 mL of 1 M HCl (e.g., pH adjustment) was added to each vial to terminate digestion. 

The digested samples were then spotted onto MALDI plates for analysis. Briefly, the machine learning model training data set consisted of 48 samples of wheat gluten pancake mix and an additional 48 samples of gluten-free mix spotted onto the MALDI targets. The scoring/evaluation data set consisted of an additional 96 spots (48 gluten-free and 48 with wheat gluten). Thus, there were 192 total spots for the MALDI analysis. Each spot was composed of 1 μL of sample and 1 μL of CHCA matrix solution.

### 2.2. Case Study 2: Salmonella enterica ATCC 51741 vs. Salmonella enterica Serovar Infantis

#### Microbial Culture and Spotting

*Salmonella enterica* (ATCC ref 0501 K derived from ATCC 51741, Microbiologics, MN 56303 USA) and *Salmonella infantis* serovar were cultured in replicates on Tryptic Soy Agar (TSA, Remel™, San Diego, CA, USA). The agar powder was dissolved in water (40 g in 1000 cc of distilled or purified water). The mixture was heated to dissolve the components fully and autoclaved for 15 min at 121 °C. Then, bacterial samples were grown on TSA agar media overnight at 37 °C. Intact samples were prepared by adding 20 μL of 20% formic acid solvent into 6 separate centrifuge tubes. Then, a 1 μL loop collected bacteria and added them to each of the 6 tubes. Three of the tubes contained the *S. enterica* samples and the remaining three contained the *S. enterica* serovar infantis. The tubes were then placed in a sonicator for 20 min to aid in cell degradation/lysis. After sonication, we vortexed each tube for 1 min on the ten setting to mix the samples thoroughly. Each of the 3 vials of *S. enterica* ATCC 51741 was spotted in 16 spot positions, totaling 48 spots. Likewise, each of the 3 vials of *S. enterica* serovar infantis was also spotted in 16 positions, totaling 48 spots. These 96 spots comprised the training set for the intact samples. An additional 96 spots from the same vials comprised the scoring set. Each spot was composed of 1 μL of sample and 1 μL of CHCA matrix solution, as described earlier.

The digested samples were then prepared by adding 20 μL of reverse osmosis water to 6 separate vials. A 1 μL loopful from the appropriate colony was then collected and added to the vials. Three of the vials contained the *S. Enterica* ATCC 51741 while the other three vials contained the *S. enterica* serovar infantis subspecies. Again, a commercially available kit was used for protein digestion (89895, Thermo Scientific, Waltham, MA, USA). In total, 20 microliters of lysis buffer and 0.75 μL of nuclease were added to each of the vials before they were placed in the sonicator for 20 min. Afterward, 25 μL of both a reduction solution and alkylation solution were added to each vial and gently mixed. The samples were then incubated for 10 min at 95 °C to reduce and alkylate proteins. We removed the samples and allowed them to cool to room temperature before 50 μL of trypsin solution was added to each vial. Each vial was then placed in an incubator at 37 °C overnight. The next day, 50 μL of digestion stop solution from the kit was added and the vials were gently mixed. Each of the 3 vials of *S. enterica* ATCC 51741 was spotted in 16 spot positions, totaling 48 spots. Likewise, each of the 3 vials of *S. enterica* serovar infantis was also spotted in 16 positions, totaling 48 spots. These 96 spots comprised the training set for the intact samples. An additional 96 spots taken from the same vials comprised the scoring set. Each spot was composed of 1 μL of the sample and 1 μL of the CHCA matrix solution described earlier.

### 2.3. Case Study 3: Presence of bla_CTX-M-65_ AMR Gene in S. enterica Serovar Infantis

#### 2.3.1. Independent Assessment of *bla_CTX-M-65_* AMR Gene Presence

The *S. enterica* serovar infantis isolates carrying the *bla_CTX-M-65_* gene were acquired from a previous study conducted in the Dominican Republic, where sample collection and *Salmonella* isolation adhered to the established project protocols [36,37,38,39]. To confirm the presence or absence of the *bla_CTX-M-65_* gene in the *S. infantis* isolates, each was first cultured in TSA (MilliporeSigma, Burlington, MA, USA) and incubated at 37 °C for 24 h. Following this, a single colony was selected from the culture plate, inoculated into Tryptic Soy Broth (TSB, MilliporeSigma, Burlington, MA, USA), and incubated overnight in a shaking incubator at 37 °C. Subsequently, DNA extraction was performed using the GenEluteTM bacterial genomic DNA kit (Sigma-Aldrich, NA2100, NA2110, or NA2120, St Louis, MO, USA), following the manufacturer’s recommended procedure. Then, the whole genome of each bacterial isolate was analyzed using an Illumina NovaSeq-6000 sequencer. Genus and species identification were conducted using FastANI (https://github.com/ParBLiSS/FastANI (accessed on 16 July 2020)) in conjunction with the Genome Taxonomy database (https://data.ace.uq.edu.au/public/gtdb/data/releases/release95/ (accessed on 16 July 2020)). Furthermore, Staramr v0.4.0 (https://github.com/phac-nml/staramr (accessed on 20 July 2020)) was employed to screen assemblies for resistance determinants by utilizing the ResFinder database from the Center for Genomic Epidemiology (CGE, https://cge.cbs.dtu.dk (accessed on 30 July 2020)) and the CGE PointFinder scheme. Herein, these results serve as the gold standard for further MALDI-TOF-based classification described below.

#### 2.3.2. Microbial Culture and Spotting

*S. enterica* serovar infantis isolates were cultured in replicates on TSA. The agar powder was dissolved in water (40 g in 1000 cc of distilled or purified water). The mixture was heated to dissolve the components fully and autoclaved for 15 min at 121 °C. Then, bacterial samples were grown on TSA media overnight at 37 °C. A single isolated colony from the agar plate was selected by using a 1 µL loop. The cell material was then mixed into 20 µL of formic acid within a sterile microcentrifuge tube. The sample was vortexed for 1 min on a setting of 10 prior to spotting on the MALDI plate. For undigested (intact) samples, three sets of samples for the MALDI-TOF analysis were created: (1) gene-positive, (2) gene-negative, and (3) scoring set samples. Positive samples contain the *bla_CTX-M-65_* AMR gene while negative samples do not. Both positive and negative sample sets were spotted 48 × 4 times. For example, each of the positive 48 samples was spotted in quadruplicate on a MALDI stainless steel plate. The gene-negative samples were also spotted in quadruplicate following the same procedure. The scoring set (evaluatory) samples were spotted in an identical fashion, with 48 × 4 of both positive and negative being used.

The same samples were also processed via a standard tryptic digestion protocol using a commercially available kit (Thermo Scientific, 89895). Several bacterial samples from multiple colonies of a culture plate were selected with a loop and diluted with 30 µL of deionized water in a centrifuge vial. Three of our nine sample vials were negative for *bla_CTX-M-65_* while six were positive. Then, vials were centrifuged at 13,000 rpm for 3 min. The supernatant was removed, leaving only the bacterial pellet. To open the cell and prepare it for digestion, we combined 50 µL of cell lysis solution in each vial. Then, 1 µL of universal nuclease was added to the mixture to neutralize the protein’s nucleic acids. A reduction solution (50 µL) was added, and, then, gently mixed with 50 µL of alkylation solution. The sample was incubated at 95 °C for 10 min to reduce and alkylate the protein. Then, 50 µL of trypsin solution was added after cooling to room temperature to facilitate protein digestion. After being incubated at 37 °C overnight, 50 µL of the digestion stop solution was added the next morning. Then, samples were spotted onto the MALDI plate. The ML training and scoring set were spotted separately. First, a training set was spotted by adding 96 total spots for the training set (36 negative and 60 positive). Twelve spots were created from each of the three gene-negative sample vials while ten spots from each vial were deposited for the six positive vials. After creating the training set, an additional 96 wells were spotted in an identical fashion to create the scoring sample set. 

For both intact and digested samples, the matrix was added shortly after placing 1 µL of the sample on the MALDI target by the addition/mixing of 1 µL of a saturated solution of alpha-cyano-hydroxycinnamic acid (CHCA) matrix solution. The solvent for this matrix was a 1:1:1 mixture of acetonitrile, ethanol, and water with 3% trifluoroacetic acid (TFA). The mixture was allowed to dry before analysis.

### 2.4. Case Study 4: Staphylococcus aureus—Enterotoxin-Positive or -Negative

#### 2.4.1. Independent Assessment of Enterotoxin Presence

In this study, *Staphylococcus aureus* isolates were obtained from a frozen stock collection at the Food Microbiology Laboratory of Texas Tech University’s School of Veterinary Medicine. These isolates were initially collected from environmental samples from a previous dairy farm project conducted in Texas. One microliter loopful was taken from each isolate and inoculated into Tryptic Soy Broth (TSB, RemelTM, San Diego, CA, USA). These cultures were incubated overnight in a shaking incubator at 200 rpm and maintained at 37 °C. Subsequently, ultra-high-temperature (UHT) milk samples were spiked with the overnight cultures and subjected to enterotoxin detection using the VIDAS^®^ Staph enterotoxin II assay (bioMérieux, Durham, NC, USA), following the manufacturer’s protocol for liquid milk samples. After the assay, the instrument automatically analyzed the results, generating a test value for each sample. This value was compared against predefined internal references (thresholds) and the results were interpreted as either enterotoxin-positive or -negative [40]. Our study used this assay as the gold standard for subsequent MALDI-TOF-based classification. 

#### 2.4.2. Microbial Culture and Spotting

Using a 1 μL loop, we collected bacteria from single isolated colonies of *S. aureus*. A total of 10 sample colonies were used, 5 enterotoxin gene-negative samples and 5 gene-positive samples. Each sample was mixed with 9 mL of Tryptic Soy Broth (TSB) and then placed into a shaking incubator at 200 rpm for 18 h. Samples were then centrifuged at maximum speed for 3 min. Once completed, the supernatant was removed from each sample, leaving only the sample pellet. The pellets were then centrifuged again for 1 min to aid in removing any remaining supernatant. The intact samples were prepared first by adding 50 μL of lysis buffer solution and 1 μL of universal nuclease to the pellet. The samples were then sent to the BioRaptor sonicator for homogenization. Once completed, the samples were split and 20 μL were taken from each gene-positive and -negative sample and placed into separate vials. In one set of vials, 20 μL of 20% formic acid was added. These samples were then vortexed on the 10 setting for 1 min. 

The second set of vials containing 20 μL were subjected to protein digestion. Again, a commercially available kit was used for protein digestion (89895, Thermo Scientific). The digested samples were prepared by adding 50 μL of lysis buffer and 1 μL of universal nuclease to each sample tube. The samples were then placed in the BioRaptor sonicator for homogenization. After, 25 μL of reduction solution and 25 μL of alkylation solution were added to each sample. The samples were incubated at 95 °C for 10 min to reduce and alkylate the proteins. They were then removed and allowed to cool to room temperature, where 50 μL of trypsin solution was added. The samples were incubated at 37 °C and 50 μL of digestion stop solution from the kit was added the next morning. The digested samples were spotted by using 48 enterotoxin-positive spots and 48 enterotoxin-negative spots. An additional 96 spots (48 positive, 48 negative) were prepared on separate slides from the same samples. Each spot was composed of 1 μL of sample and 1 μL of CHCA matrix.

### 2.5. MALDI-TOF-MS Analysis

Data were acquired using a Shimadzu Axima Performance MALDI-TOF mass spectrometer. For both intact and digested samples, the laser power was set at 74 (arbitrary units) and 50 Hz repetition rate. The relationship between the arbitrary instrument unit for laser power and true irradiance or peak power is unknown. An automated raster pattern was used for the 500 profiles collected, with 2 pulses per profile. For intact samples, with a mass range of 500–20,000 *m*/*z* in the linear TOF, positive ion mode was employed. For digested samples, with a mass range of 200–7000 *m*/*z* in the reflection HiRes TOF, positive ion mode was employed. After a MALDI spectrum was acquired, the Axima Performance operating software was used to identify centroid mass peaks and record peak list data to a comma-delimited (.csv) text file. Peak lists contained the *m*/*z* value and intensity automatically assigned by the software. The instrument was calibrated with a TOFMIX synthetic peptide mixture of known molecular weights (LaserBioLabs). The MALDI-TOF response with molecular weight was investigated using known synthetic peptides of specific molecular weights and concentrations. Peptide Mix 1 (Angiotensin II (1046.2 Da), Angiotensin I (1296.5 Da), Neurotensin (1672.9 Da), ACTH [1–17] (2093.5 Da), ACTH [18–39] (2465.7 Da)); Peptide Mix 3 (Bovine Insulin Chain B (3495.9 Da), Bovine Insulin (5733.6 Da), Aprotinin (6511.5 Da), Ubiquitin Bovine (8564.8 Da)); and protein calibration standards (Horse Heart Cytochrome C (12,360.1 Da), Horse Myoglobin (16,951.5 Da), Trypsinogen (23,980.9 Da), Yeast Enolase (46,670.9 Da), Bovine Serum Albumin (66,429.9 Da)) from LaserBioLabs were employed.

### 2.6. Data Mining

The peak list files were then formatted into bins of prescribed width using a LabView VI written in-house. Binning is a crucial aspect of the machine learning workflow and is described pictorially in Figure 1B. Essentially, the mass spectrum is divided into bins of finite width and observed peaks of *m*/*z* within a tolerance are grouped together within a bin. Typically, our bins are named to identify a certain central mass; however, the bin name is essentially a label (as opposed to a numerical value). Binning introduces an artificial loss of mass spectral resolution but is essential to ensure patterns in data are conserved. Binning parameters were altered for different mass ranges based upon experimentally observed variance in peak *m*/*z* assigned for identical peaks in the mass spectrum. The guiding principle is to ensure the bin width is wider than the variability of peak *m*/*z* assigned in the software so that identical peaks always are grouped into the same bin.

Bins were defined between *m*/*z* = 500–1500, 1500–2500, 2500–7000, 7000–12,000, 12,000–20,000 Da for intact samples. Bins were defined between *m*/*z* = 200–999, 1000–1999, 2000–2999, 3000–3999, 4000–4999, 5000–6999 Da for the digested samples. The bin widths (tolerances) for the intact files in each mass range were: 1.25, 2.2, 3.5, 8, and 35 Da and the bin widths for the digested files for each mass range were all 0.5 Da. Then, after a MALDI-TOF spectrum was acquired, the Axima Performance operating software was used to identify centroid mass peaks and record data to a comma-delimited (.csv) text file. The mass for bins for the intact files varied (*δ* M) by 1, 2, 5, 10, and 50 Da for each mass range. The delta mass (*δ* M) for the digested files were all fixed at 1 Da. After formatting all files, mass spectral data were compiled in a spreadsheet and transposed into the format needed for data mining.

All data mining was accomplished within RapidMiner Studio software (Version 9.10, RapidMiner GmbH). The gradient-boosted tree was the only machine learning model that was utilized since the results of an earlier study within our laboratory provided clear data showing that this method performed best with MALDI-TOF data sets [20]. There were two dataset spreadsheets for every case study for the gradient-boosted tree machine-learning model. First, a training dataset spreadsheet was used to build the machine learning model. For all samples in the training set, the true state (negative or positive), as defined by the gold standard method, was identified as the ‘label’ within the software. In addition, binned MALDI-TOF mass spectra for each sample were associated with the labels on each line of the spreadsheet. When the machine learning model runs, the known training set data is used to draw statistical inferences between the presence of MALDI-TOF peaks at certain *m*/*z* values and the true diagnostic outcome. The patterns resulting from these computations become the machine learning model. Then, a second spreadsheet is built containing MALDI-TOF mass spectra and sample identifiers. This second spreadsheet is composed of different spectra collected from different spots on the MALDI target and forms the scoring data set. The machine learning model constructed is then applied to the scoring dataset and the model makes a prediction in a dichotomous manner (positive or negative). Then, we compare the prediction to reality for each sample within this scoring set to predict sensitivity and specificity. This allows for the evaluation of performance. The gradient-boosted model has its own variables, which can be adjusted by the user within the software. The gradient-boosted tree model has many adjustable parameters, including the number of trees, number of bins, and learning rate. During the investigative trials, the number of trees was varied between 50, 101, 401, and 701; the number of bins was varied as being either 20 or 40; and the learning rate was varied among the values: 0.01, 0.0001, 0.00001. Again, upon each run, the training data set was used to construct the model and the separate scoring data set was used to evaluate. Each run consisted of the data from the appropriate case study. Diagnostic sensitivity (sens) and specificity (spc), as well as the Youden Index (J), were computed for all of the case studies and samples, as described in Equations (1)–(3).

## 3. Results

### 3.1. Estimate of Sensitivity Increase via Tryptic Digestion

**Figure 2:**
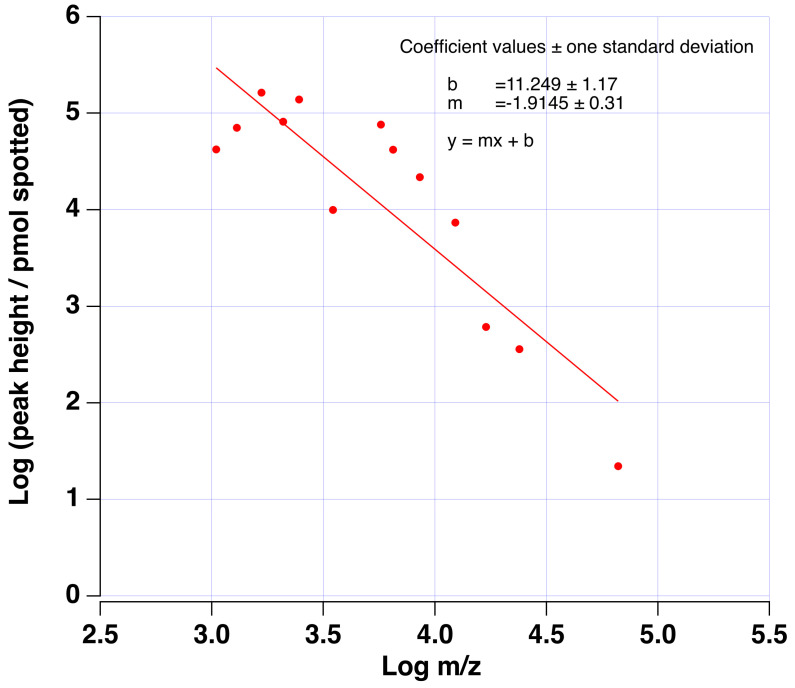
Plot of the Log of the mass normalized MALDI-TOF signal (peak height) vs. the Log *m*/*z* for a known mass of a series of known peptides and proteins spotted. The slope of the best-fit line suggests a nearly 2-Log reduction in signal per decade molecular weight (MW). The data quantify the MALDI-TOF response with increasing molecular weight.

Signal intensity in MALDI–TOF is known to be attenuated as molecular weight increases. Figure 2 reports the signal intensity loss we observe as a function of *m*/*z* for a series of known peptides and proteins that were spotted on a MALDI plate in a known quantity. As observed, the mass-normalized signal intensity loss of nearly two log units per decade of molecular weight (MW) was observed as a rough quantitative estimate of this effect. Such loss in signal (sensitivity) severely limits the ability to achieve diagnostic sensing via MALDI TOF since only abundant, lower-molecular-weight proteins may routinely be observed during routine screening of samples.

**Figure 3:**
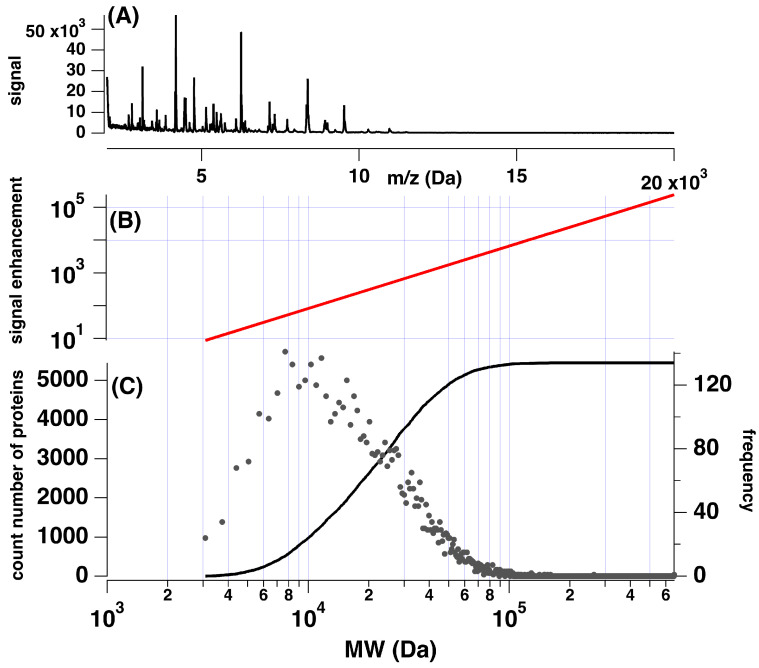
(**A**) Typical MALDI mass spectrum of a bacterial isolate. Note—large number of peaks present in the low mass region and low frequency of peaks > 10 kDa. (**B**) Predicted MALDI-TOF signal enhancement for protein digest vs. molecular weight relative to a 1 kDa peptide based upon the results of Figure 2. (**C**) Count number (-) of known proteins in *Salmonella enterica* plotted vs. molecular weight and frequency or abundance (•) vs. molecular weight. Despite ~80% of known proteins having mass >10 kDa, few are observed within MALDI-TOF spectra, as demonstrated in (**A**).

To fully understand the impact of this, consider the known proteome of *Salmonella enterica*, as reported by the UniProt database [41]. Uniprot lists 5445 known proteins of mass > 3 kDa encoded by the genome of this bacterium. Figure 3C reports the count number and abundance of these known, intact proteins as a function of molecular weight (Da). As observed, proteins near 10 kDa appear to be most common for *Salmonella* and roughly 80% of proteins associated with *Salmonella* are above 10 kDa in mass. However, the MALDI-TOF spectra of this organism (Figure 3A) typically exhibit only ≤3–10 peaks in the higher mass region of the spectrum. Despite the vast majority (number count) of proteins present in *Salmonella* being >10 kDa, very few peaks appear in a MALDI mass spectrum within that mass range. Many of the larger, low-abundance proteins that escape detection may be of diagnostic value to identifying the bacteria and/or classifying samples into sub-categories but are undetected in a typical experiment on intact proteins.

As protein molecular weight increases, it becomes far less likely that adequate signal-to-noise will be observed in a MALDI-TOF mass spectrum to detect and record the presence of such proteins. Since proteolytic digestion generally yields peptide fragments in the mass range of 700–1500 Da, a range of masses more optimal for sensitive detection in MALDI-TOF-based workflows is achieved [42]. Thus, reducing the molecular weight of proteins while maintaining discriminatory molecular characteristics may allow improved diagnostics via MALDI-TOF sensing with machine learning.

The pattern observed from the data and presented in Figure 2 allows for the estimation of the MALDI–TOF signal enhancement achieved through the digestion of proteins as a function of molecular weight. The expected enhancement factor computed from the trend observed is reported in Figure 3B. For this enhancement estimate, we consider a prototypical 1 kDa peptide resulting from the proteolytic digestion of a larger protein. For a parent protein of only 3–4 kDa, a signal enhancement of approx. 10 may be expected for the detection of the smaller peptide fragment. However, larger signal enhancements of 100–10,000-fold would be expected when the parent protein is more than 10–100 kDa. Since the proteolytic digestion of proteins enhances the detection of potential biomarkers, such experimental steps may affect machine-learning-based sorting.

### 3.2. Results for Case Studies

#### 3.2.1. Case Study 1: Pancake Mix—Gluten-Free vs. Wheat Gluten Mix

**Figure 4:**
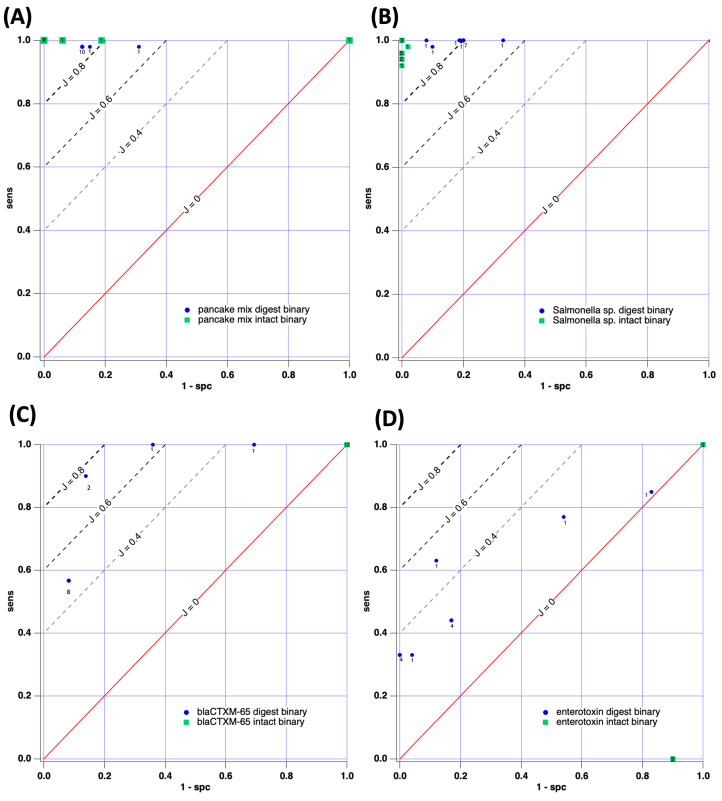
ROC curves for the machine-learning-based sorting of digested and intact samples when binary peak intensity is used. (**A**) Case Study 1: pancake mixes; (**B**) Case Study 2: *Salmonella* speciation; (**C**) Case Study 3: *bla_CTX-M-65_* gene detection; (**D**) Case Study 4: Enterotoxin-positive or -negative. The small numeral adjacent to each data point is the number of trials this value was observed in.

**Figure 5:**
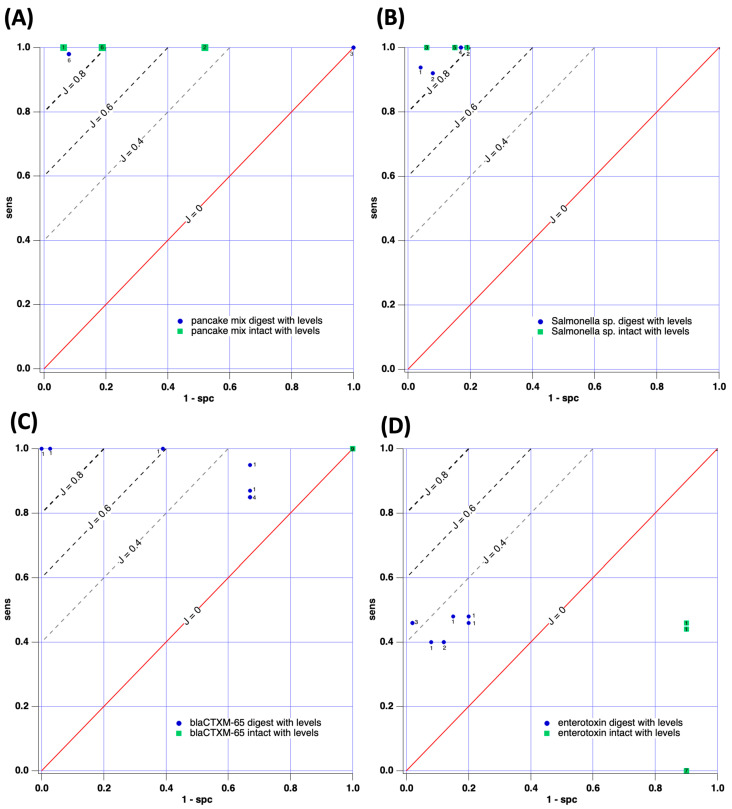
ROC curves for the machine-learning-based sorting of digested and intact samples when continuous peak intensities were used. (**A**) Case Study 1: pancake mixes; (**B**) Case Study 2: *Salmonella* speciation; (**C**) Case Study 3: *bla_CTX-M-65_* gene detection; (**D**) Case Study 4: Enterotoxin-positive or -negative. The small numeral adjacent to each data point is the number of trials this value was observed in.

The initial case study involved attempts to distinguish between pancake mix with and without wheat gluten present (e.g., gluten-free). Since the chemical composition of these two samples is fundamentally different, we assign this case study a relatively low level of difficulty in terms of machine learning/sorting. Figure 4A and Figure 5A report receiver operating characteristic (ROC) curves describing the performance of the machine learning models for intact (green data points) and digested (blue data points) samples. Tables of performance data are also included in the manuscript’s Supplement S1 Section.

Results for the binary peak quantitation method are observed in Figure 4A; both intact and digested samples of pancake mixes were easily distinguishable when the binary method of peak quantitation was used. Generally, the Youden index was J > 0.8 and J = 1 was obtained in several instances (perfect sorting of scoring set). The average *J* value for the digested samples was 0.838 while for the intact samples, the mean J was 0.896. This result indicates that intact samples performed slightly better in classification by machine learning; however, no significant difference between the average J values for digested vs. intact data sets was observed (*p* < 0.05) for this case study. The ratio between the average J values (J_dig_/J_intact_) was 0.93, indicating similar performance in sorting between intact and digested cases. In short, for Case Study 1, in which sample composition is believed to be quite different, no advantage was gained by conducting the protein digestion procedure. It should also be noted that the average J values for both intact and digested samples (0.75 and 0.56), when peak intensity values were included in the machine learning model, yielded poorer performance than the simple binary approach. This outcome is counter-intuitive but regularly encountered. We posit the decrease in performance occurs because of the large sample-to-sample signal intensity irreproducibility that is encountered in MALDI (up to 80% RSD by our estimates).

#### 3.2.2. Case Study 2: *Salmonella enterica* ATCC 51741 vs. *Salmonella enterica* Serovar Infantis

The second case study aimed to sort two types of *Salmonella* based on MALDI-TOF and machine learning. ROC curves describing the results are presented in Figure 4B for binary peak analysis and 5B when peak intensities were used. In both cases, high-performing sorting was achieved, with J values typically > 0.8 indicated. The average J observed for digested samples was 0.81 and the average was J = 0.96 for sorting intact samples when binary peak intensity information was used. In this instance, a significant difference in performance was observed between intact and digested samples, with intact performing significantly better. Again, the ratio between the average J values (J_dig_/J_intact_) was 0.85, indicating that both sample preparation methods sorted the serovars well. When peak intensities were included in the analysis, the mean J for the digested samples was 0.852; J = 0.875 for the intact samples and J_dig_/J_intact_ = 0.97. For this case, the mean *J* values are statistically indistinguishable. In Case Study 2, no advantage of digestion was demonstrated and both methods of sample preparation led to the effective sorting of samples (similar to Case Study 1).

#### 3.2.3. Case Study 3: *bla_CTX-M-65_* Gene-Positive or -Negative

The third case study aimed to sort a scoring set of *Salmonella* samples known to be either positive or negative for the *bla_CTX-M-65_* gene. This gene encodes for the production of a 283 amino acid β-lactamase responsible for catalyzing the hydrolysis/destruction of antibiotics containing the lactam ring. The gene’s presence infers the bacterial ability to potentially be resistant to a wide palette of common antibiotics, including penams, cephalosporins, cephamycins, monobactams, carbapenems, and carbacephems. After the development of the ML algorithm through analysis of the training set, as described in the methods section, a scoring set of separate samples was used to generate the ROC curves reported in Figure 4C and Figure 5C. Again, intact and digested samples were directly compared for sorting success. In the case of Figure 4C, all intact protein trials (12 trials) yielded no ability to sort gene-positive from gene-negative under the conditions tested. However, digested samples achieved more success, with *J* ranging from 0.3 to 0.784. In total 11/12 data points yielded J > 0.48 for the digested samples. The performance for sorting the digested samples was significantly better than the intact samples (*p* < 0.05) when binary peak descriptors were used.

Figure 5C illustrates results for *bla_CTX-M-65_* gene detection when peak levels/intensities were used in the ML model. Again, the ML model exhibited no ability to sort positives from negatives for intact samples under the conditions used. However, digested samples yielded more favorable *J* values ranging from 0.18 to 1. Yet, two-thirds of the trials attempted yielded J < 0.4 and only marginal diagnostic performance was achieved. Nevertheless, Case Study 3 demonstrated, conclusively, that digestion of the protein present during sample preparation can lead to significantly improved sorting via MALDI-TOF-based ML workflows.

#### 3.2.4. Case Study 4: *Staphylococcus aureus*–Enterotoxin-Positive or -Negative

Case Study 4 aimed to group bacteria based on the indication (or lack of) of enterotoxin through the VIDAS^®^ SET2 assay. Enterotoxins are heat-stable, water-soluble proteins secreted by *S*. *aureus* and known to cause episodes of food poisoning in humans [43]. Figure 4D reports the ROC analysis for both intact and digested samples when binary peak descriptors were used. As observed, the intact samples (green) could not be correctly identified by the machine learning models tested. However, J values as large as 0.51 were observed for samples digested prior to MALDI-TOF. Most (10/12) samples exhibited J ≈ 0.3, reflecting only a marginal ability to differentiate between organisms capable of producing enterotoxin and those that are not. When considering ROC curves when peak intensities are used (Figure 5D), again, protein-intact samples offered no diagnostic value for the models tested. The digested samples performed better, yielding J = 0.26–0.44 for the trials. This level of performance as a diagnostic is poor and alternative tests, such as immunological or genetic-based options, are higher performing. Despite the limited diagnostic success, digested samples yielded statistically better performances (J values) compared to intact samples for Case Study 4.

## 4. Discussion

**Figure 6:**
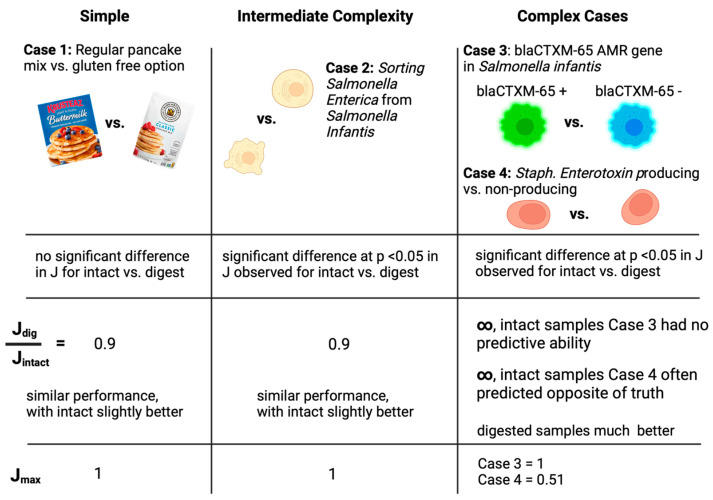
Summary of results for case studies. Case Studies 1–4 were of increasing complexity/difficulty. The figure reports whether statistically different values in mean J were observed (*p* < 0.05) between intact and digest samples (the ratio J_dig_/J_intact_ is the ratio between average J values for digested vs. intact samples) and the maximum J encountered for the set of samples. ML models for Case Study 1 (pancake mixes) yielded excellent sorting for both digested and intact samples, with Youden indices (J) near one and insignificant differences in J-values between methods. Case Study 2 results suggested intact analysis yielded significantly better J values with *p* < 0.05; however, both models were largely effective at classifying, as evidenced by a J ratio of 0.9. In Case Studies 3 and 4, significant differences were observed in J values, with digested samples performing much better. Intact samples for Case Studies 3 and 4 illustrated no predictive ability within the ML models.

Table 1 and Figure 6 summarize the average results for the diagnostic sensitivity, specificity, and Youden index for this study. A rapid study of the J values leads to a summary conclusion that intact samples performed slightly better in Case Studies 1 and 2. However, in Case Studies 3 and 4, intact samples offered no diagnostic ability while digested samples offered some ability to sort into dichotomous groups. We argue that Case Studies 3 and 4 (AMR gene presence and enterotoxin presence) represent more challenging cases for sorting since the samples are more fundamentally similar in composition. The outcome that protein digestion for more complex samples dramatically improves diagnostic sorting represents a crucial development in the field of MALDI-TOF machine learning workflows. Future practice may incorporate digestion steps as part of the workflow to enhance molecular diagnostics for challenging cases. Conversely, when samples are believed to be quite chemically dissimilar (as in Case Studies 1 and 2), digestion steps may unnecessarily complicate the workflow and induce time-consuming and expensive steps that are unnecessary for success. Thus, the results of our analysis suggest protein digestion prior to MALDI-TOF machine learning is only particularly valuable to analysts when confronted with challenging cases of sorting.

A second remarkable outcome of experimentation is the realization that including MALDI-TOF signal intensity values often yields poorer diagnostic performance. Formatting data as binary peak list files (peak present or not at certain *m*/*z*) often yields improved results. This outcome is largely counter-intuitive. We believe this originates due to the large spot-to-spot variability in signal intensity observed in MALDI-TOF measurements, which can exceed 80% relative standard deviation. This large instrumental variability coupled with biological variability in protein expression, differential ion suppression within the mass spectrometer, and irreproducibility in the extent of protein digestion likely creates sufficient ‘noise’ encoded within signal intensities to render this information useless for pattern recognition. We should also comment that this condition may be overcome if improved sample preparation/sample spotting steps are used.

## 5. Conclusions

In conclusion, this research paper showcases the significance of protein digestion in enhancing sensitivity and specificity in MALDI-TOF-based bacterial identification and molecular diagnostics. The integration of machine learning algorithms with protein digestion offers great promise in disease diagnosis and biomarker discovery when samples are adequately complex. Guidance for best practice for sample preparation remains complex and an art relying on the skill of the analyst. When samples vary considerably, protein digestion adds costly and unnecessary steps. However, when only minor differences are present in the sample chemistry, protein digestion and MALDI-TOF analysis on the resultant sample can offer transformative improvement in diagnostic accuracy. These findings present compelling evidence for the continued exploration, development, and use of this innovative approach to transform clinical microbiology, diagnosis, and personalized medicine.

## Figures and Tables

**Table 1:** Table reporting the average sensitivity, specificity, Youden index (J), and maximum J for case studies and trials.

Case Study	Trial	Sens (Avg.)	Spc (Avg.)	J_avg_	J_max_
Case Study 1	Intact binary	1	0.90	0.90	1
	Intact w/signals	1	0.75	0.75	0.94
	Digest binary Digest w/signals	0.98 0.99	0.86 0.61	0.84 0.60	0.86 0.90
Case Study 2	Intact binary	0.97	0.99	0.96	1
	Intact w/signals	1	0.88	0.88	0.94
	Digest binary	0.99	0.81	0.81	0.92
	Digest w/signals	0.98	0.86	0.84	0.90
Case Study 3	Intact binary	1	0	0	0
	Intact w/signals	1	0	0	0
	Digest binary	0.70	0.82	0.51	0.78
	Digest w/signals	0.91	0.51	0.42	1
Case Study 4	Intact binary	0.33	0.07	−0.60	0
	Intact w/signals	0.1	0.1	−0.80	−0.4
	Digest binary	0.47	0.82	0.29	0.51
	Digest w/signals	0.44	0.90	0.34	0.44

## Data Availability

Data are contained within this article and/or Supplementary Material.

## Supplementary Materials

The following supporting information can be downloaded at: https://www.mdpi.com/article/10.3390/s23198042/s1, Table S1: Results of all trials.
