# Supplementary material for: Effect of Tryptic Digestion on Sensitivity and Specificity in MALDI-TOF-Based Molecular Diagnostics through Machine Learning
Source: Sensors (Basel). 2023 Sep 23;23(19):8042. doi: 10.3390/s23198042 (PMC10575185; doi:10.3390/s23198042)
Supplement: Supplementary file 1 [file sensors-23-08042-s001.zip › sensors-2626987-supplementary.pdf]

SUPPLEMENT S1: Results of Individual Trials.

Note: Number of trees, number of bins, learning rate are user adjustable parameters within the RapidMiner machine learning software associated with the gradient boosted tree model used in this work.

Case Study 1 – Gluten vs Gluten Free Pancake Mix. Tables of machine learning predictive results.

Intact Samples with Peak Intensity used.

# of Trees	# of Bins	Learning Rate	Sensitivity	Specificity	J	Diagnostic value
101	40	0.00001	1	0.8125	0.8125	excellent
101	40	0.0001	1	0.8125	0.8125	excellent
101	40	0.01	1	0.9375	0.9375	excellent
401	40	0.00001	1	0.8125	0.8125	excellent
401	40	0.0001	1	0.8125	0.8125	excellent
401	40	0.01	1	0.48	0.48	marginal
701	40	0.00001	1	0.8125	0.8125	excellent
701	40	0.0001	1	0.8125	0.8125	excellent
701	40	0.01	1	0.48	0.48	marginal

Intact Samples using binary (0,1) values for peak / no peak.

# of Trees	# of Bins	Learning Rate	Sensitivity	Specificity	J	Diagnostic value
50	20	0.01	1	1	1	excellent
50	20	0.0001	1	1	1	excellent
50	20	0.00001	1	1	1	excellent
101	20	0.01	1	1	1	excellent
101	20	0.0001	1	0	0	None
101	20	0.00001	1	1	1	excellent
401	20	0.01	1	0.94	0.94	excellent
401	20	0.0001	1	1	1	excellent
401	20	0.00001	1	1	1	excellent
701	20	0.01	1	0.8125	0.8125	excellent
701	20	0.0001	1	1	1	excellent
701	20	0.00001	1	1	1	excellent

Digested Samples with Peak Intensity used.

# of Trees	# of Bins	Learning Rate	Sensitivity	Specificity	J	Diagnostic value
101	40	0.00001	0.98	0.92	0.9	excellent
101	40	0.0001	1	0	0	None
101	40	0.01	1	0	0	None
401	40	0.00001	0.98	0.92	0.9	excellent
401	40	0.0001	0.98	0.92	0.9	excellent
401	40	0.01	0.98	0.92	0.9	excellent
701	40	0.00001	0.98	0.92	0.9	excellent
701	40	0.0001	0.98	0.92	0.9	excellent
701	40	0.01	1	0	0	None

Digested Samples using binary (0,1) values for peak / no peak.

# of Trees	# of Bins	Learning Rate	Sensitivity	Specificity	J	Diagnostic value
50	20	0.01	0.98	0.875	0.855	excellent
50	20	0.0001	0.98	0.875	0.855	excellent
50	20	0.00001	0.98	0.875	0.855	excellent
101	20	0.01	0.98	0.875	0.855	excellent
101	20	0.0001	0.98	0.875	0.855	excellent
101	20	0.00001	0.98	0.875	0.855	excellent
401	20	0.01	0.98	0.85	0.83	excellent
401	20	0.0001	0.98	0.875	0.855	excellent
401	20	0.00001	0.98	0.875	0.855	excellent
701	20	0.01	0.98	0.69	0.67	good
701	20	0.0001	0.98	0.875	0.855	excellent
701	20	0.00001	0.98	0.875	0.855	excellent

Case Study 2 – *Salmonella Enterica* and *Salmonella Infantis*

Intact Samples with Peak Intensity used.

# of Trees	# of Bins	Learning Rate	Sensitivity	Specificity	J	Diagnostic value
101	40	0.00001	1	0.85	0.85	excellent
101	40	0.0001	1	0.81	0.81	excellent
101	40	0.01	1	0.94	0.94	excellent
401	40	0.00001	1	0.85	0.85	excellent
401	40	0.0001	1	0.85	0.85	excellent
401	40	0.01	1	0.94	0.94	excellent
701	40	0.00001	1	0.85	0.85	excellent
701	40	0.0001	1	0.85	0.85	excellent
701	40	0.01	1	0.94	0.94	excellent

Intact Samples using binary (0,1) values for peak / no peak.

# of Trees	# of Bins	Learning Rate	Sensitivity	Specificity	J	Diagnostic value
50	20	0.01	0.98	0.98	0.96	excellent
50	20	0.0001	0.94	1	0.94	excellent
50	20	0.00001	0.92	1	0.92	excellent
101	20	0.01	1	1	1	excellent
101	20	0.0001	0.96	1	0.96	excellent
101	20	0.00001	0.96	1	0.96	excellent
401	20	0.01	1	1	1	excellent
401	20	0.0001	0.96	1	0.96	excellent
401	20	0.00001	0.94	1	0.94	excellent
701	20	0.01	1	1	1	excellent
701	20	0.0001	0.96	1	0.96	excellent
701	20	0.00001	0.96	1	0.96	excellent

Digested Samples with Peak Intensity used.

# of Trees	# of Bins	Learning Rate	Sensitivity	Specificity	J	Diagnostic value

101	40	0.00001	0.9375	0.96	0.8975	excellent
101	40	0.0001	1	0.83	0.83	excellent
101	40	0.01	0.92	0.92	0.84	excellent
401	40	0.00001	1	0.8125	0.8125	excellent
401	40	0.0001	1	0.83	0.83	excellent
401	40	0.01	1	0.83	0.83	excellent
701	40	0.00001	1	0.8125	0.8125	excellent
701	40	0.0001	0.92	0.92	0.84	excellent
701	40	0.01	1	0.83	0.83	excellent

Digested samples using binary (0,1) values for peak / no peak.

# of Trees	# of Bins	Learning Rate	Sensitivity	Specificity	J	Diagnostic value
50	20	0.01	0.98	0.9	0.88	excellent
50	20	0.0001	1	0.8	0.8	excellent
50	20	0.00001	1	0.8	0.8	excellent
101	20	0.01	1	0.92	0.92	excellent
101	20	0.0001	1	0.8	0.8	excellent
101	20	0.00001	1	0.8	0.8	excellent
401	20	0.01	1	0.8125	0.8125	excellent
401	20	0.0001	1	0.8	0.8	excellent
401	20	0.00001	1	0.81	0.81	excellent
701	20	0.01	1	0.67	0.67	good
701	20	0.0001	1	0.8	0.8	excellent
701	20	0.00001	1	0.8	0.8	excellent

Case Study 3 – *Salmonella Infantis* blaCTXM-65 Gene ML results.

Intact Samples with Peak Intensity used.

# of Trees	# of Bins	Learning rate	Sensitivity	Specificity	J	Diagnostic value
101	40	0.00001	1	0	0	None
101	40	0.0001	1	0	0	None
101	40	0.01	1	0	0	None
401	40	0.00001	1	0	0	None
401	40	0.0001	1	0	0	None
401	40	0.01	1	0	0	None
701	40	0.00001	1	0	0	None
701	40	0.0001	1	0	0	None
701	40	0.01	1	0	0	None

Intact Samples using binary (0,1) values for peak / no peak.

# of Trees	# of Bins	Learning rate	Sensitivity	Specificity	J	Diagnostic value
50	20	0.01	1	0	0	None
50	20	0.0001	1	0	0	None
50	20	0.00001	1	0	0	None

101	20	0.01	1	0	0	None
101	20	0.0001	1	0	0	None
101	20	0.00001	1	0	0	None
401	20	0.01	1	0	0	None
401	20	0.0001	1	0	0	None
401	20	0.00001	1	0	0	None
701	20	0.01	1	0	0	None
701	20	0.0001	1	0	0	None
701	20	0.00001	1	0	0	None

Digested Samples with Peak Intensity used.

# of Trees	# of Bins	Learning rate	Sensitivity	Specificity	J	Diagnostic value
101	40	0.00001	0.85	0.33	0.18	marginal
101	40	0.0001	0.85	0.33	0.18	marginal
101	40	0.01	1	1	1	excellent
401	40	0.00001	0.85	0.33	0.18	marginal
401	40	0.0001	0.87	0.33	0.20	marginal
401	40	0.01	1	0.972	0.972	excellent
701	40	0.00001	0.85	0.33	0.18	marginal
701	40	0.0001	0.95	0.33	0.28	marginal
701	40	0.01	1	0.61	0.61	good

Digested samples using binary (0,1) values for peak / no peak.

# of Trees	# of Bins	Learning rate	Sensitivity	Specificity	J	Diagnostic value
50	20	0.01	0.9	0.861	0.761	excellent
50	20	0.0001	0.567	0.917	0.484	marginal
50	20	0.00001	0.567	0.917	0.484	marginal
101	20	0.01	0.867	0.917	0.784	excellent
101	20	0.0001	0.567	0.917	0.484	marginal
101	20	0.00001	0.567	0.917	0.484	marginal
401	20	0.01	1	0.64	0.64	good
401	20	0.0001	0.567	0.917	0.484	marginal
401	20	0.00001	0.567	0.917	0.484	marginal
701	20	0.01	1	0.306	0.306	marginal
701	20	0.0001	0.567	0.917	0.484	marginal
701	20	0.00001	0.567	0.917	0.484	marginal

Case Study 4 – *Staphylococcus* Toxin producing vs. No Toxin.

Intact Samples with Peak Intensity used.

# of Trees	# of Bins	Learning rate	Sensitivity	Specificity	J	Diagnostic value
101	40	0.00001	0	0.1	-0.9	None

101	40	0.0001	0	0.1	-0.9	None
101	40	0.01	0	0.1	-0.9	None
401	40	0.00001	0	0.1	-0.9	None
401	40	0.0001	0	0.1	-0.9	None
401	40	0.01	0.46	0.1	-0.44	None
701	40	0.00001	0	0.1	-0.9	None
701	40	0.0001	0	0.1	-0.9	None
701	40	0.01	0.44	0.1	-0.46	None

Intact Samples using binary (0,1) values for peak / no peak.

# of Trees	# of Bins	Learning Rate	Sensitivity	Specificity	J	Diagnostic value
50	20	0.01	0	0.1	-0.9	None
50	20	0.0001	0	0.1	-0.9	None
50	20	0.00001	0	0.1	-0.9	None
101	20	0.01	1	0	0	None
101	20	0.0001	1	0	0	None
101	20	0.00001	0	0.1	-0.9	None
401	20	0.01	0	0.1	-0.9	None
401	20	0.0001	0	0.1	-0.9	None
401	20	0.00001	0	0.1	-0.9	None
701	20	0.01	1	0	0	None
701	20	0.0001	0	0.1	-0.9	None
701	20	0.00001	0	0.1	-0.9	None

Digested Samples with Peak Intensity used.

# of Trees	# of Bins	Learning Rate	Sensitivity	Specificity	J	Diagnostic value
101	40	0.00001	0.48	0.8	0.28	marginal
101	40	0.0001	0.46	0.98	0.44	marginal
101	40	0.01	0.4	0.92	0.32	marginal
401	40	0.00001	0.48	0.85	0.33	marginal
401	40	0.0001	0.46	0.98	0.44	marginal
401	40	0.01	0.4	0.88	0.28	marginal
701	40	0.00001	0.46	0.8	0.26	marginal
701	40	0.0001	0.46	0.98	0.44	marginal
701	40	0.01	0.4	0.88	0.28	marginal

Digested samples using binary (0,1) values for peak / no peak.

# of Trees	# of Bins	Learning Rate	Sensitivity	Specificity	J	Diagnostic value
50	20	0.01	0.33	0.96	0.29	marginal
50	20	0.0001	0.44	0.83	0.27	marginal
50	20	0.00001	0.33	1	0.33	marginal
101	20	0.01	0.63	0.88	0.51	marginal
101	20	0.0001	0.44	0.83	0.27	marginal
101	20	0.00001	0.33	1	0.33	marginal
401	20	0.01	0.77	0.46	0.23	marginal

401	20	0.0001	0.44	0.83	0.27	marginal
401	20	0.00001	0.33	1	0.33	marginal
701	20	0.01	0.85	0.17	0.02	poor
701	20	0.0001	0.33	1	0.33	marginal
701	20	0.00001	0.44	0.83	0.27	marginal
